# Semaphorin6A acts as a gate keeper between the central and the peripheral nervous system

**DOI:** 10.1186/1749-8104-2-28

**Published:** 2007-12-18

**Authors:** Olivier Mauti, Elena Domanitskaya, Irwin Andermatt, Rejina Sadhu, Esther T Stoeckli

**Affiliations:** 1 Developmental Neuroscience, Institute of Zoology, University of Zurich, Winterthurerstrasse 190, 8057 Zurich, Switzerland; 2 Novartis, DOC, Lichtstrasse 35, CH-4002 Basel, Switzerland

## Abstract

**Background:**

During spinal cord development, expression of chicken SEMAPHORIN6A (SEMA6A) is almost exclusively found in the boundary caps at the ventral motor axon exit point and at the dorsal root entry site. The boundary cap cells are derived from a population of late migrating neural crest cells. They form a transient structure at the transition zone between the peripheral nervous system (PNS) and the central nervous system (CNS). Ablation of the boundary cap resulted in emigration of motoneurons from the ventral spinal cord along the ventral roots. Based on its very restricted expression in boundary cap cells, we tested for a role of Sema6A as a gate keeper between the CNS and the PNS.

**Results:**

Downregulation of Sema6A in boundary cap cells by *in ovo *RNA interference resulted in motoneurons streaming out of the spinal cord along the ventral roots, and in the failure of dorsal roots to form and segregate properly. PlexinAs interact with class 6 semaphorins and are expressed by both motoneurons and sensory neurons. Knockdown of PlexinA1 reproduced the phenotype seen after loss of Sema6A function both at the ventral motor exit point and at the dorsal root entry site of the lumbosacral spinal cord. Loss of either PlexinA4 or Sema6D function had an effect only at the dorsal root entry site but not at the ventral motor axon exit point.

**Conclusion:**

Sema6A acts as a gate keeper between the PNS and the CNS both ventrally and dorsally. It is required for the clustering of boundary cap cells at the PNS/CNS interface and, thus, prevents motoneurons from streaming out of the ventral spinal cord. At the dorsal root entry site it organizes the segregation of dorsal roots.

## Background

During development of the nervous system, axons navigate long distances to connect to their targets. Along their trajectories they encounter a large variety of guidance cues that support their navigation [1,2]. Axon guidance cues are subdivided into long-range and short-range guidance cues. They belong to a relatively small number of protein families, the immunoglobulin superfamily of cell adhesion molecules [3], the Eph/ephrins [4], the netrins [5,6], the semaphorins [7,8] and their receptors, plexins and neuropilins [9,10]. More recently, morphogens such as Wnts and Shh have also been implicated in axon guidance [11-13].

The semaphorins comprise a large family subdivided into eight subclasses based on structural criteria and their expression in vertebrates or non-vertebrate organisms [7,14,15]. Class 1 and 2 semaphorins are expressed only in invertebrates, classes 3, 4, 6, and 7 are expressed only in vertebrates, class 5 semaphorins are expressed in both invertebrates and vertebrates, whereas class V consists of a viral semaphorin. With respect to their function, soluble class 3 semaphorins are the best characterized. They have been shown to act mainly as repellents but, in some cases, also as attractants for extending axons. Class 3 semaphorins bind to a receptor complex composed of Neuropilin-1 or -2 and a member of the class A plexins [10,15], although there is at least one exception to this rule [16].

Plexins are expressed in a highly dynamic pattern during development of the nervous system [17-20]. They are subdivided into four classes comprising a total of nine members in mammals and seven members in chicken [20]. Plexins of class A and B were shown to bind to transmembrane semaphorins in the absence of neuropilins [21,22]. PlexinBs are receptors for class 4 semaphorins, whereas PlexinAs were shown to be receptors for class 6 semaphorins [22-25]. Interestingly, transmembrane semaphorins have functions in axon guidance and synapse formation that are independent of neuropilins [22]. The long cytoplasmic tail of Sema6A contains binding sites for Ena/VASP-like protein EVL and may, therefore, directly regulate cytoskeletal dynamics [26]. Consistent with these structural features, Sema6A was suggested to act as a receptor [27], similar to findings for Sema1a, the closest ortholog of Sema6A in invertebrates [28]. Sema1a was shown to act both as a repellent [29,30] and as an attractant [31]. A receptor function for Sema1a was reported in the visual system of *Drosophila*, where photoreceptor cells depended on Sema1a for their targeting to the optic lobe [32].

In mammals, Sema6A was shown to affect pathfinding of thalamocortical axons [27] and to be required for cell migration in the cerebellum [33]. The mode of action has not been determined in these studies but, based on the expression pattern and the analysis of the phenotypes, a repulsive mechanism has been suggested in the latter. This would be consistent with *in vitro *studies that demonstrated a repulsive role of Sema6A on sympathetic axons [22,34]. More recently, a repellent activity of Sema6D on proprioceptive sensory afferents has been shown in both mouse and chicken [25]. The targeting of proprioceptive axons was dependent on PlexinA1 mediating the repulsive activity of Sema6C/D. PlexinA1 was also shown to be the binding partner of Sema6D in neural crest cell migration during heart development [23]. In these studies a receptor function of Sema6D was demonstrated [24]. Thus, transmembrane class 6 semaphorins are bifunctional molecules in axon guidance and cell migration. They can act as a ligand for PlexinAs but also transmit a signal themselves.

In vertebrates, the receptors for Sema6A in cerebellar development have not been identified. However, *in vitro *binding studies have indicated that Sema6A can bind to PlexinA2 and A4 [22], whereas Sema6B binds to PlexinA1and A4 [22], Sema6C was suggested to bind to PlexinD1 [35], and finally Sema6D was shown to bind to PlexinA1 in neural crest cell migration [23,24].

Analysis of SEMA6A expression during chicken spinal cord development revealed its restriction to the ventral ventricular zone, the origin of oligodendrocytes, and, most strikingly, to cells at the ventral motor axon exit point (VMEP) and the dorsal sensory axons entry point. Cells located at the transition zone between the PNS and the CNS were shown to have gate keeper function [36,37]. In analogy to their function they are called boundary cap cells (BCCs). BCCs are derived from a late migrating population of neural crest cells [38]. They express Krox20 and the 1E8 antigen in addition to the more general neural crest marker Sox10. BCCs are necessary to prevent emigration of motoneurons from the ventral spinal cord [37]. More recently, the boundary cap was identified as a source of neural crest stem cells that give rise to glia and sensory neurons of the dorsal root ganglion (DRG) [39-41].

Here, we show that Sema6A is required for the gate keeper function of BCCs, as in the absence of Sema6A BCCs fail to cluster properly at the CNS/PNS interface and, thus, cannot prevent the emigration of motoneurons in a PlexinA1-dependent manner. At the dorsal root entry site Sema6A is required for the appropriate segregation of dorsal roots.

## Results

### SEMA6A is expressed in boundary cap cells

**Figure 1:**
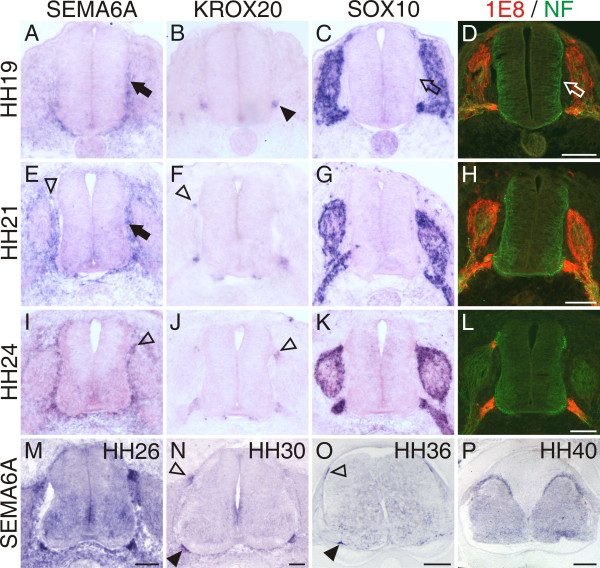
SEMA6A is expressed in neural crest cells that give rise to boundary cap cells. **(a) **Neural crest cells that give rise to boundary cap cells express SEMA6A while they are still migrating ventrally (HH19; arrow). Boundary cap cells start to cluster first at the VMEP. **(b) **Only those cells that have formed clusters at the VMEP express the BCC marker KROX20 (arrowhead). The neural crest markers **(c) **SOX10 and the **(d) **1E8 epitope are expressed by many neural crest-derived cell populations and are not restricted to BCCs at HH19. Note that neither SOX10 (open arrow in (c)), nor 1E8 (open arrow in (d)) are expressed in BCCs while they are still migrating. First clusters of BCCs next to the DREZ marked by **(e) **SEMA6A expression (open arrowhead) or **(f) **KROX20 (open arrowhead) are detectable at lumbosacral levels by HH21. At this stage, many SEMA6A-expressing cells are still migrating along the neural tube to reach the ventral BCC cluster (arrow in (e)). **(i-l) **A similar situation is found at HH24. SEMA6A expression is clearly detectable in dorsal BCCs (open arrowhead in (i); compare with (j)). **(n, o) **After HH30, SEMA6A expression in dorsal (open arrowhead in (n)) and ventral (arrowhead in (n)) BCCs decreased but was still visible by HH36 (o). *In situ *hybridizations on adjacent transverse sections of the lumbosacral spinal cord are shown for SEMA6A (a, e, i, m-p), KROX20 (b, f, j), and SOX10 (c, g, k) at HH19 (a-d), HH21 (e-h), HH24 (i-l) as indicated. Sections shown in (d, h, l) were stained for 1E8 (red) and neurofilament (green). Bars are 100 μm in (a-n), 200 μm in (o), and 500 μm in (p).

SEMA6A shows a much more restricted expression pattern during development of the chicken spinal cord compared to the mouse. In contrast to the mouse, where SEMA6A was found throughout the ventral spinal cord and in DRGs [22], it was expressed only transiently in the ventral spinal cord but never in DRGs in the chick. Most striking, however, was its expression in cells at the boundary between the CNS and the PNS (Figure 1). SEMA6A was detectable in a ventral stream of neural crest cells at stage 19 (HH19; Figure 1a) [42]. At that time, small clusters of BCCs identified by KROX20 [43] were seen only at the VMEP (Figure 1b). Motor axons start to leave the spinal cord shortly before the cluster of BCCs is detectable by 1E8 staining (data not shown) [37,44]. Clustering of BCCs at the dorsal root entry site started at HH21, as visualized by KROX20 (Figure 1f). By HH24, BCC clusters were very prominent both ventrally at the VMEP and dorsally at the dorsal root entry zone (DREZ; Figure 1j). BCCs expressed SEMA6A (Figure 1a,e,i,m–o), KROX20 (Figure 1b,f,j), SOX10 (Figure 1c,g,k) [45], the 1E8 antigen (an epitope of P0; Figure 1d,h,l) [46], and Cadherin-7 (data not shown) [47]. The SEMA6A-expressing cells were not SOX10 or 1E8 positive while they migrated toward the VMEP. Similarly, the expression of KROX20 was visible only after cells had clustered. After clustering, boundary cap cells were positive for SOX10 (Figure 1c,g,k) and 1E8 (Figure 1d,h,l). SOX10 and 1E8 were not restricted to BCCs but were also expressed by Schwann cells associated with the ventral roots and in DRGs. Thus, SEMA6A is the earliest marker for cells that end up in clusters at the boundary between the CNS and the PNS.

### Sema6A is required to keep motoneurons from migrating out of the ventral spinal cord

**Figure 2:**
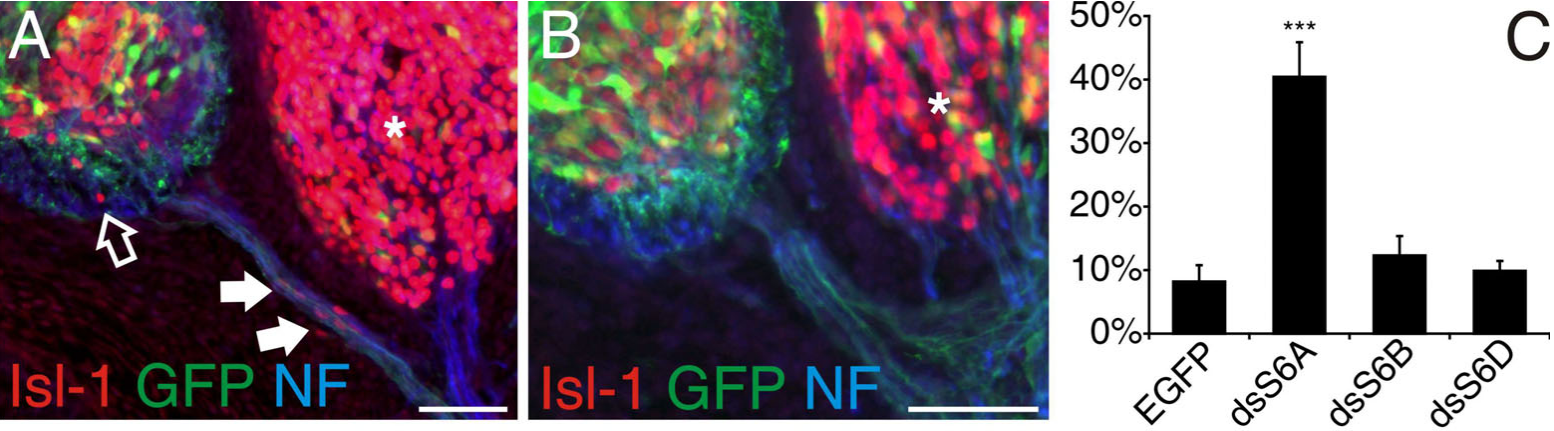
Downregulation of Sema6A in BCCs results in translocation of motoneurons out of the spinal cord. **(a) **In the absence of Sema6A from BCCs, motoneurons stream out of the ventral spinal cord and migrate along the ventral roots (arrows). The open arrow points to a motoneuron that is located in the ventral funiculus. **(b) **In control-treated embryos motoneurons along ventral roots or in the ventral funiculus were rarely seen. Motoneurons were identified by Isl-1 (red). An EGFP plasmid was co-injected with the dsRNA derived from SEMA6A. Axons were stained with an antibody against neurofilament (blue). Note that sensory neurons in the DRG (asterisk in (a, b)) are also stained by Isl-1. **(c) **Perturbation of Sema6B or Sema6D did not enhance the number of motoneurons in the periphery compared to control-treated embryos injected only with the plasmid encoding EGFP. Three asterisks indicate *P *< 0.0001 for the comparison between dsS6A and all other treatment groups. Values are given as mean ± standard error of mean. Bar: 50 μm.

BCCs at the VMEP were shown to prevent the emigration of motoneurons from the ventral spinal cord [37]. The failure in BCC cluster formation after ablation of neural crest cells resulted in streams of motoneurons migrating along the axons of the ventral root. Because of the restricted expression of SEMA6A in BCCs, we set out to test whether Sema6A would be required for the role of BCCs as gate keepers between the CNS and the PNS. To this end, we used *in ovo *RNA interference (RNAi), our previously established method to induce loss-of-function phenotypes [48]. *In ovo *RNAi at HH12-14 efficiently targeted neural crest cells and resulted in downregulation of Sema6A but did not interfere with the expression of other family members of class 6 semaphorins (data not shown). Downregulation of Sema6A did indeed reproduce the phenotype seen after ablation of the BCCs (Figure 2) [37]. Groups of motoneurons identified by Isl-1 staining were found along the ventral roots in all HH25 embryos lacking Sema6A function (Figure 2a). Motoneurons exiting the spinal cord were seen, on average, in 40% of the sections from the lumbosacral spinal cord (range 25–54%). Single motoneurons leaving the ventral spinal cord were occasionally detected in control embryos (Figure 2b). However, cells did not emigrate in clusters as seen after downregulation of Sema6A, and the number of sections that contained motoneurons along the ventral roots was much smaller in control-treated embryos compared to embryos lacking Sema6A function. Downregulation of the other class 6 semaphorins, Sema6B (12%) and Sema6D (10%), did not significantly enhance emigration of motoneurons compared to control embryos expressing enhanced green fluorescent protein (EGFP; 8%). SEMA6D but not SEMA6B was found to be expressed in BCCs (IA and ES, unpublished observation). However, as seen for KROX20, expression started only after clustering of BCCs. SEMA6D was not found in BCCs that were still migrating (data not shown).

### Sema6A is required for appropriate entry of sensory afferents into the dorsal spinal cord

**Figure 3:**
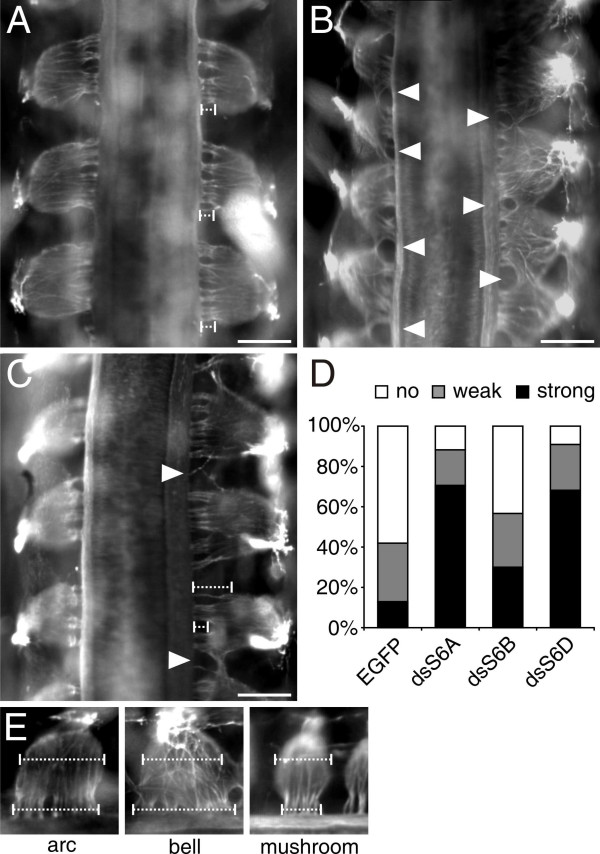
Lack of Sema6A and Sema6D in dorsal BCCs results in aberrant segregation of dorsal roots. **(a) **In control embryos axon bundles from each dorsal root ganglion extend to the DREZ in a well organized manner. Roots from adjacent DRGs are segregated and they are all of the same length (dashed bars). **(b) **In contrast, in embryos lacking Sema6A, roots from adjacent DRGs are no longer segregated (arrowheads). The arrangement of roots arising from individual DRGs is strongly disorganized and roots are often formed by fibers from two adjacent DRGs (arrowheads in (b)). **(c) **Similarly, roots are disorganized in embryos lacking Sema6D (arrowheads). In addition the length of the roots varied more in the absence of Sema6D (compare dashed bars in (c)). **(d) **Strong phenotypes were seen in 71% of the embryos lacking Sema6A and in 68% of the embryos lacking Sema6D. Only 13% of the embryos injected with an EGFP plasmid had a comparable phenotype. Downregulation of Sema6B resulted in aberrant DRG shapes and root arrangement in 30% of the embryos. **(e) **The shapes of DRGs were classified as arc-like when the distance between the most anterior and the most posterior fiber emanating from the DRG was the same as the anteroposterior diameter of the DRG; as bell-shaped when the fibers spread an anteroposterior length that was bigger than the diameter of the DRG; and as mushroom-like when the fibers entered the dorsal spinal cord in a segment that was shorter than the diameter of the DRG. Note that the diameter of the mushroom-like DRGs was smaller than the diameter of arc-like or bell-shaped DRGs. Bar: 200 μm.

The strong effect on motoneurons and the fact that SEMA6A was expressed also in BCCs at the DREZ prompted us to analyze the effect of Sema6A downregulation on sensory afferents. Loss of Sema6A in dorsal BCCs had a severe effect on the arrangement of dorsal roots (Figure 3). In control embryos analyzed at HH25/26, fibers emanating from a single DRG formed, on average, four to five well separated fiber bundles or roots that entered the dorsal spinal cord via the DREZ. Roots derived from neighboring DRGs were clearly segregated (Figure 3a). This was not the case after downregulation of Sema6A in dorsal BCCs (Figure 3b). In 71% of these embryos the arrangement of dorsal roots and their number were severely perturbed (Figure 3d). Furthermore, the shape of the DRGs was more variable than in control embryos, including many DRGs with a bell shape; that is, with a distance between the most anterior and the most posterior fiber entering the spinal cord that was larger than the anteroposterior size of the DRG (Figure 3e). In control embryos these two lengths were identical, resulting in an arc-like shape of the DRG. In addition to the embryos exhibiting a strong phenotype, we found 18% with a weak phenotype (Figure 3d). In these embryos no bell-shaped DRGs were found despite the fact that the number and arrangement of roots varied. In more than 70% of the embryos lacking Sema6A in BCCs, we found no segregation between adjacent DRGs; that is, roots were formed by fibers emanating from two adjacent DRGs. Only 12% of the embryos treated with double-stranded RNA (dsRNA) derived from SEMA6A were normal. In 58% of the control-treated embryos, DRGs and their roots were normal (Figure 3a,d). Only 13% of them exhibited a strong phenotype.

Interestingly, in contrast to our findings at the VMEP, downregulation of Sema6D resulted in a dorsal phenotype (Figure 3c,d). Embryos lacking Sema6D were, overall, not much different from embryos lacking Sema6A. In only 9% of the embryos were arrangement and number of dorsal roots normal. Sixty-eight percent of the embryos exhibited a strong phenotype, and 23% a weak phenotype. Downregulation of Sema6B resulted in a qualitatively different phenotype. Despite the fact that DRGs exhibited a mushroom-like shape (Figure 3e), the number and the arrangement of the roots were much less affected (data not shown; Figure 3d).

### PlexinAs, known receptors for Sema6A, are expressed by motoneurons and sensory neurons

**Figure 4:**
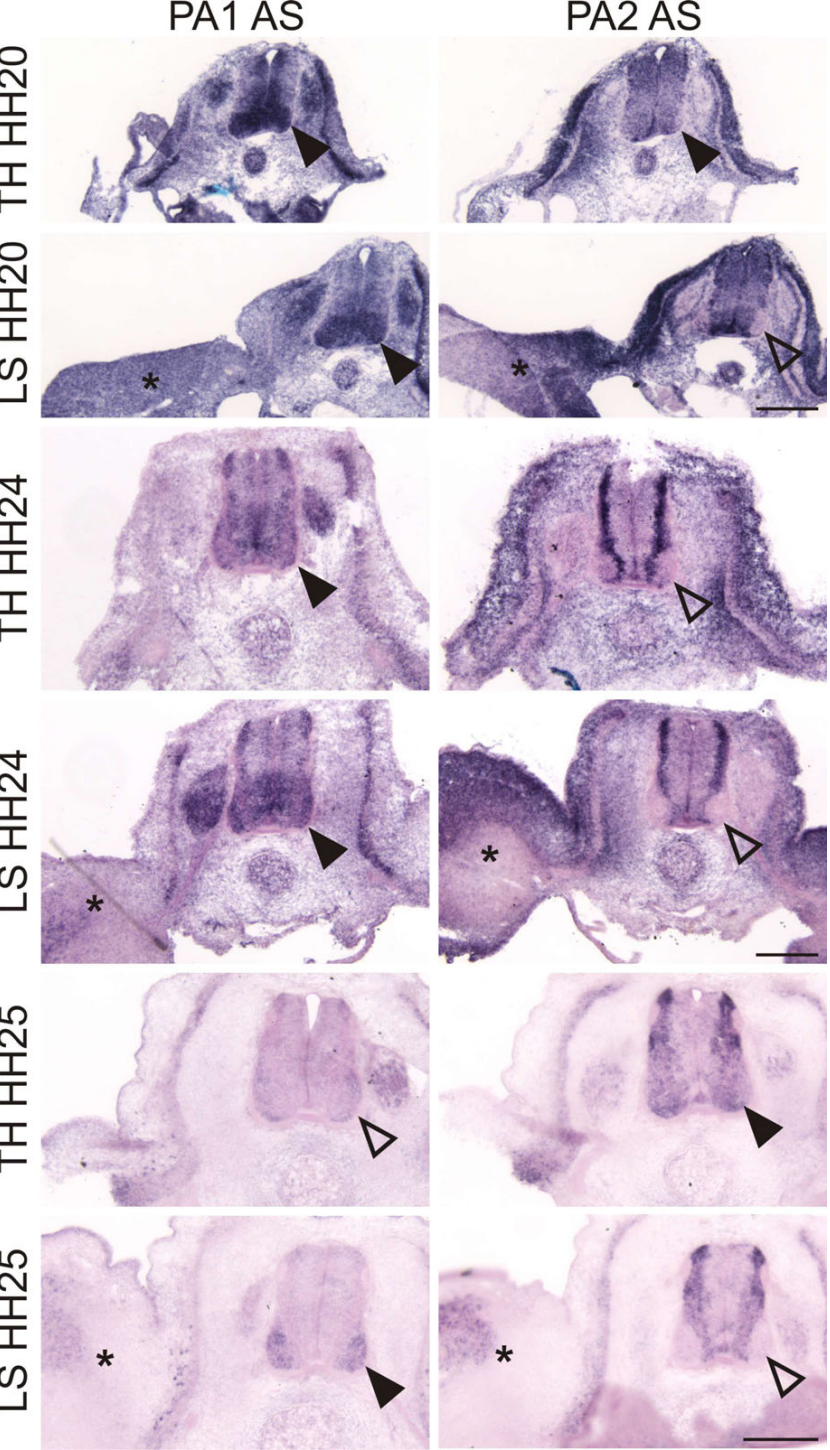
The expression of PLEXINA1 (PA1) and PLEXINA2 (PA2) differs between the thoracic and the lumbosacral levels of the spinal cord. Based on their expression pattern, none of the PlexinAs can be excluded as a binding partner for Sema6A [20]. In addition to the dynamic changes over time, the expression of PLEXINA1 and PLEXINA2 differs strongly between thoracic and lumbosacral levels of the spinal cord. PLEXINA1 is strongly expressed in the ventral spinal cord at HH20, but remains to be expressed strongly only at the lumbosacral but not the thoracic level at HH24 and HH25. Even more pronounced are the changes of PLEXINA2 expression. At HH20, expression is detectable in lateral motoneurons only at the thoracic but not at the lumbosacral level of the spinal cord. This difference is even more pronounced at older stages. AS, antisense probe; TH, thoracic level; LS lumbosacral level. Arrowheads indicate expression of either PA1 or PA2; open arrowheads indicate no or very weak expression. Asterisks label the hind limb to indicate that sections were taken from the lumbosacral level of the spinal cord. Bar: 200 μm.

PlexinAs were shown to act both as ligands and as receptors for class 6 semaphorins [23-25]. Previously, we had shown that the expression patterns of chicken PLEXINAs were highly dynamic both in motoneurons and sensory neurons [20]. Based on these analyses, all PlexinAs were potential binding partners for Sema6A. PLEXINA1 was expressed at high levels in the ventral spinal cord and in DRGs at HH18 to HH22 [20] (Figure 4). PLEXINA2 was expressed in the lumbosacral spinal cord at HH18 but was subsequently downregulated in motoneurons during development. Expression in DRGs was weak between HH20 and HH30. In contrast, PLEXINA4 was virtually not expressed in the spinal cord at HH18 but was strongly upregulated in motoneurons at HH22. PLEXINA4 was also expressed in DRGs at HH22 and later stages [20]. Based on the temporal and spatial expression pattern, none of the PlexinAs could be ruled out as a binding partner for Sema6A at the VMEP and at the dorsal root entry site. PLEXINA2 was the least likely candidate because we focused our analysis on the lumbosacral level of the spinal cord, where PLEXINA2 was already below detection levels by HH20, in contrast to the thoracic level where PLEXINA2 remained expressed.

**Figure 5:**
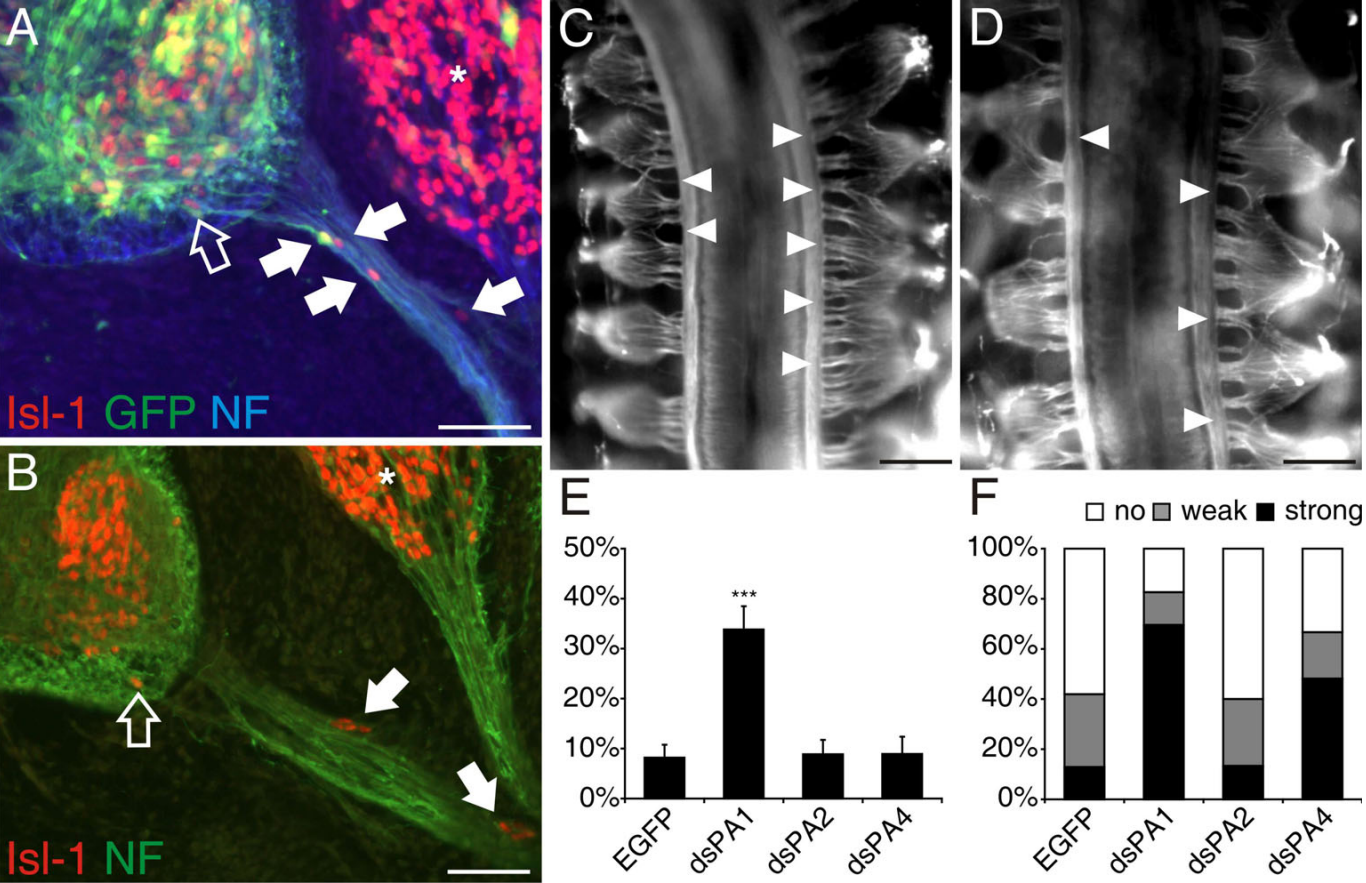
Downregulation of PlexinA1 results in the same phenotype as seen in the absence of Sema6A. **(a, b) **Motoneurons streaming out of the ventral spinal cord identified by Isl-1 staining were only found after downregulation of PlexinA1 (arrows). The open arrow points to a motoneuron that is located in the ventral funiculus. Note that sensory neurons in the DRG (asterisk) are also stained by Isl-1. **(e) **Lack of none of the other PlexinAs enhanced the number of motoneurons found along the ventral roots compared to control-treated embryos (*p *= 0.0001 for the comparison between dsPA1 and all other treatment groups (indicated by three asterisks); values are given as mean ± standard error of the mean; see Figure 2b). **(c, d) **The phenotype seen after downregulation of Sema6A in dorsal BCCs was mimicked by both lack of PlexinA1 (c) and PlexinA4 (d). The effects of PlexinA downregulation were qualitatively different, however. In the absence of PlexinAs, the arrangement of DRGs, and not only the arrangement of their roots, was disorganized. **(f) **A phenotype was seen in 83% of embryos lacking PlexinA1 and in 67% of the embryos lacking PlexinA4. Bar 50 μm in (a, b); 200 μm in (c, d).

We first knocked down PlexinAs in motoneurons using *in ovo *RNAi. For each PlexinA we used two independent long dsRNAs (see Materials and methods). Downregulation was specific for the targeted gene (Additional file 1). Consistent with its strong expression in motoneurons at the time when they extend their axons out of the VMEP, we found pronounced effects after downregulation of PlexinA1. In 34% of the sections from the lumbosacral region that we analyzed we found groups of motoneurons along the ventral root (Figure 5). All embryos lacking PlexinA1 were affected and had motoneurons outside the spinal cord in 13–52% of the sections taken from the lumbosacral spinal cord. Thus, the phenotype observed after RNAi for PLEXINA1 was qualitatively and quantitatively comparable to the phenotype observed after RNAi for SEMA6A (compare Figures 5a,b,e and 2a,c). Downregulation of PlexinA2 and A4 had no effect on the migratory behavior of motoneurons. The number of motoneurons outside the spinal cord was not different from control (Figure 5e). We counted ectopic motoneurons in 9% of the lumbosacral sections from embryos lacking PlexinA2 or PlexinA4 compared to 8% for control-treated embryos.

Next we analyzed the effect of PlexinA downregulation at the dorsal root entry site. In the absence of PlexinA1 and PlexinA4 (Figure 5c,d), we found phenotypes that resembled those seen after downregulation of Sema6A and Sema6D (Figure 3b,c). Downregulation of PlexinA1 perturbed dorsal root formation and segregation in the vast majority of the embryos. Only 17% of the embryos had normal DRGs (Figure 5f). Seventy percent of them exhibited a strong phenotype. Detailed analysis of the embryos lacking PlexinA1 revealed that the phenotype was qualitatively different from the phenotype seen in the absence of Sema6A. In addition to fusions of adjacent DRGs, we found a different type of DRG shape to predominate in embryos lacking PlexinA1. DRGs were narrower than normal and had a reduced number of roots. The distance between the most anterior and the most posterior fiber emanating from a single DRG was much shorter than the width of the DRG. Therefore, we qualified these DRGs as mushroom-like (Figure 3e). Variable shapes of DRGs were found after loss of PlexinA4 function, where 48% of the embryos exhibited a strong phenotype. In both cases it was sometimes not possible to identify individual DRGs, as they were fused across spinal cord segments. In the absence of PlexinA1, only 17% of the embryos had normal DRGs, and in the absence of PlexinA4, only 33% had normal DRGs. Downregulation of PlexinA2 did not show an effect on dorsal root arrangement; 60% of the embryos were normal. Aberrant root arrangement and mushroom-shaped DRGs were only found in 13% of the embryos.

### Sensory but not motor axons are repelled by Sema6A

**Figure 6:**
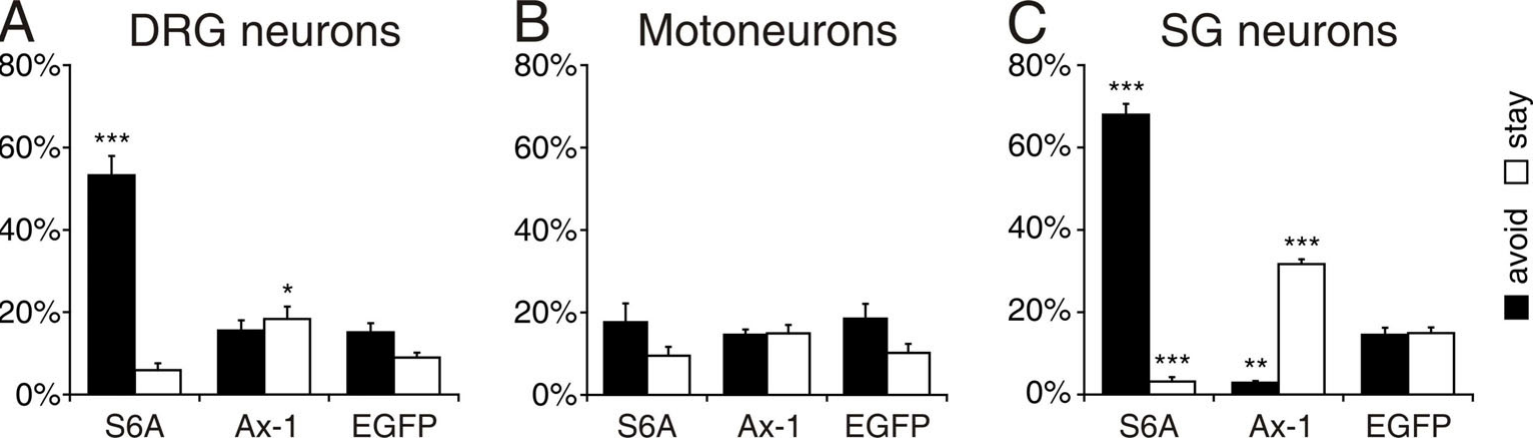
Axons of DRG and sympathetic neurons but not motor axons are repelled by Sema6A. **(a) **Upon encountering a COS cell expressing Sema6A, 53% of all DRG axons were found to react with avoidance, that is, they turned away from the cell or stopped rather than growing onto the Sema6A-positive COS cell (*p *= 0.0002 for the comparison between S6A/Ax-1 and S6A/EGFP (indicated by three asterisks)). COS cells expressing Axonin-1 (Ax-1) were perceived as slightly more attractive than control COS cells expressing only EGFP (*p *= 0.006 for S6A/Ax-1 and 0.02 for S6A/EGFP (indicated by asterisk)). **(b) **Motor axons were found to be indifferent to all types of COS cells. The majority showed neither attraction nor repulsion when encountering Sema6A or Axonin-1 compared to EGFP-expressing COS cells. **(c) **The majority of sympathetic axons (68%) were avoiding Sema6A-expressing cells (*p *< 0.0001 (three asterisks)). Compared to DRG axons and motor axons, sympathetic axons were more strongly attracted by Axonin-1-expressing COS cells (*p *< 0.0001 for Ax-1/S6A and Ax-1/EGFP). This is reflected by the fact that avoidance of Axonin-1-expressing cells was significantly lower compared to EGFP-expressing cells (*p *= 0.003 (two asterisks)). Similarly, axons of sympathetic neurons (SG) were significantly less attracted to Sema6A-expressing compared to EGFP-expressing cells (*p *< 0.0001 (three asterisks)). Values are given as mean ± standard error of the mean.

To get a lead on the mechanism of Sema6A function in boundary control, we turned to an *in vitro *assay (Figure 6). We wanted to assess whether Sema6A in BCCs had an attractive or a repulsive effect on sensory and motor axons, respectively. For this purpose, we transfected COS cells with SEMA6A and used them as a substrate for DRG neurons and motoneurons. We also used sympathetic neurons as they were shown to react to Sema6A contact with growth cone collapse [34]. Axonin-1 was used as a control protein. We scored the behavior of axons encountering transfected COS cells as 'repulsion' when axon failed to grow onto a transfected cell by either stopping or turning away. The score was 'attraction' when axons readily crossed from a non-transfected to a transfected COS cell but did not cross back from the transfected to a non-transfected cell. Axons that readily crossed from a non-transfected to a transfected cell and back to a non-transfected cell were scored as 'crossing', or, in other words, were considered not to be affected by the protein expressed on COS cells. COS cells expressing EGFP were used as an additional control to measure the 'baseline behavior' of axons growing on COS cells. As expected, few cells reacted with repulsion or attraction to COS cells expressing EGFP. For all types of neurons, we found that more than 70% of the axons crossed EGFP-transfected COS cells readily (Table 1). The behavior was different in response to COS cells expressing Sema6A. Both DRG (Figure 6a) and sympathetic axons (Figure 6c) avoided Sema6A-positive cells. The effect was stronger for sympathetic neurons, where avoidance was found for 68% of the axons compared to 53% of the DRG axons (Table 1). Axons of motoneurons did not show a reaction to either Sema6A or Axonin-1 that differed from the behavior on EGFP-expressing cells (Figure 6b). Interestingly, we found that significantly more sympathetic axons reacted with attraction to Axonin-1 than to control COS cells expressing EGFP (Figure 6c). In conclusion, axons of DRG neurons were repelled by Sema6A, whereas motor axons did not react at all to Sema6A.

**Table 1:** DRG and sympathetic axons avoid Sema6A-expressing COS cells

	Sema6A			Axonin-1			EGFP		
			
	Avoid (%)	Stay (%)	Cross (%)	Avoid (%)	Stay (%)	Cross (%)	Avoid (%)	Stay (%)	Cross (%)
DRG	53.3 ± 4.7	5.9 ± 1.6	40.8 ± 4.7	15.5 ± 2.5	18.3 ± 3.0	66.1 ± 4.2	15.1 ± 2.2	9.0 ± 1.2	75.9 ± 1.7
MN	17.6 ± 4.6	9.5 ± 2.1	72.9 ± 4.0	14.6 ± 1.3	14.9 ± 2.1	70.5 ± 3.0	18.5 ± 3.6	10.2 ± 2.2	71.3 ± 1.4
SG	68.0 ± 2.6	3.1 ± 1.1	28.9 ± 3.4	2.9 ± 0.4	31.7 ± 1.1	65.4 ± 1.2	14.5 ± 1.7	14.9 ± 1.4	70.6 ± 3.1

COS cells transfected with Sema6A, Axonin-1, or EGFP were used as substrate for DRG, motor, and sympathetic neurons. For each condition at least 200 axons from 2 (sympathetic) or 3 (sensory and motoneurons) independent experiments were counted. Values represent mean ± standard error of the mean. MN, motoneuron; SG sympathetic neuron.

### The effect of Sema6A in PNS/CNS border control is caused by a defect in BCC clustering

**Figure 7:**
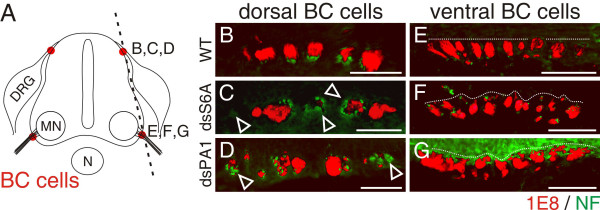
Clustering of BCCs is perturbed in the absence of Sema6A and PlexinA1. **(a-g) **Longitudinal sections of HH25 spinal cords (as indicated by the dashed line in (a)) were stained with 1E8 (red) and anti-neurofilament antibodies (green) to analyze dorsal (b-d) and ventral (e-g) BCCs from untreated embryos (b, e) or embryos treated with dsRNA derived from SEMA6A (c, f) and PLEXINA1 (d, g), respectively. Dorsal BCC clusters in control embryos (b) were relatively homogenous in size, closely aligned with the roots, and regularly spaced. In contrast, in the absence of either Sema6A (c) or PlexinA1 (d), the size of BCC clusters was very variable and their arrangement was highly disorganized. Many axons were not in contact with BCCs at all or only with individual cells or microclusters (open arrowheads in (c, d)). Ventral BCC clusters were smaller than their dorsal counterparts even in control embryos (e). Therefore, the effect of Sema6A (f) or PlexinA1 (g) perturbation on cluster size was less obvious. However, the absence of Sema6A and PlexinA1 clearly disrupted the alignment of ventral BCC clusters (compare dashed lines in (e) with (f, g)). The color of the axons stained with anti-neurofilament antibodies and visualized with an Alexa350-coupled secondary antibody was changed to green using Adobe Photoshop CS2 to get better contrast. EGFP used to select the appropriate sections is not shown. MN, motoneurons; N, notochord. Bar 100 μm.

To gain insight into the mechanism of Sema6A function as a gate keeper, we analyzed the formation of BCC clusters in the absence of Sema6A from migrating neural crest cells (Figure 7). BCC clusters were reduced in size or missing altogether in the absence of Sema6A and their localization along the anteroposterior and the dorsoventral axes of the spinal cord was perturbed. The aberrant arrangement of BCC clusters was detectable both dorsally at the DREZ (Figure 7c) and ventrally at the VMEP (Figure 7f). At the dorsal root entry site 1E8-positive cells were no longer found in regular, dense clusters, as seen in control-treated embryos (Figure 7b). Many axons were not in close contact with BCCs in the absence of Sema6A (Figure 7c). At the VMEP BCC clusters were smaller than their dorsal counterparts (compare Figure 7e and 7b). The downregulation of Sema6A in ventral BCCs resulted in their aberrant clustering along both the anteroposterior and the dorsoventral axes.

**Figure 8:**
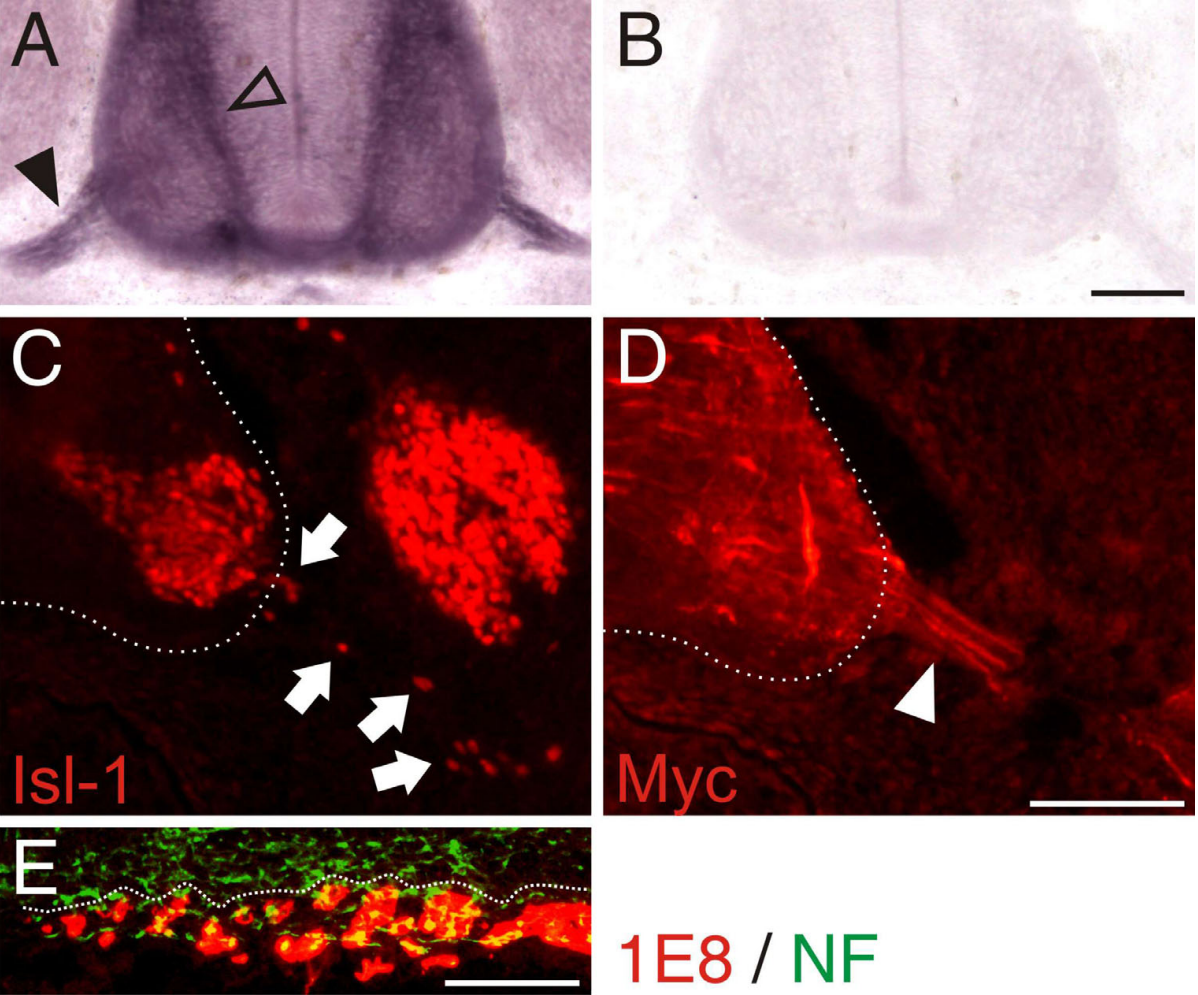
Ectopic expression of the Sema6A ectodomain or full-length Sema6A in motoneurons competes with BCC-derived Sema6A binding to motoneurons. The AP-tagged ectodomain of Sema6A binds to axons expressing PlexinAs. **(a) **Both commissural axons (open arrowhead) and motor axons (arrowhead) express PlexinAs [20] and bind the Sema6A ectodomain. **(b) **No binding of the AP-tag alone was detectable. **(c) **Ectopic expression of both the ectodomain of Sema6A (not shown) and the full-length myc-tagged form resulted in motoneurons streaming out of the spinal cord along the ventral roots (arrows). **(d) **Staining of the myc tag demonstrates expression of Sema6A in motor axons (arrowhead), consistent with a competitive role of motor axon-derived Sema6A with BCC-derived Sema6A in the periphery. **(e) **As seen after downregulation of either Sema6A in BCCs (compare to Figure 7f) or PlexinA1 in motoneurons (compare to Figure 7g), ectopic expression of Sema6A resulted in the aberrant formation of BCC clusters. Bar: 100 μm.

Similarly, downregulation of PlexinA1 in sensory (Figure 7d) and motoneurons (Figure 7g) resulted in the same aberrant arrangement of BCC clusters as seen after interference with Sema6A expression. As none of the PlexinAs was expressed in BCCs [20] and no homophilic interaction of Sema6A was found *in vitro *(data not shown), we concluded that Sema6A on BCCs was necessary to recognize a stop signal on sensory and motor axons. This signal was likely provided by PlexinA1, as axons were not decorated with 1E8-positive BCCs in the absence of it (Figure 7d). In support of this hypothesis, we found binding of AP-tagged Sema6A to both commissural and motor axons but not to BCCs, in accordance with the expectation that Sema6A would bind only to PlexinA-expressing cells and not to Sema6A-expressing cells (Figure 8a).

As an alternative approach to block the interaction between PlexinAs on motor axons and Sema6A on BCCs, we expressed the ectodomain or full-length Sema6A in motoneurons, where normally no Sema6A is found in chick (except for a transient expression at HH26; Figure 1m). Providing Sema6A on motor axons would prevent PlexinA1 from interacting with Sema6A on BCCs because it would compete with BCC-derived Sema6A. BCC clusters would thus fail to form properly due to the absence of the stop signal (Figure 8e) and motoneurons would stream out of the spinal cord at the VMEP just as found after either Sema6A downregulation in BCCs or PlexinA1 downregulation in motoneurons. This is indeed what we observed (Figure 8c).

In summary, our results support the hypothesis that Sema6A on BCCs interacts with PlexinA1 on motor axons to recognize the VMEP, where BCCs aggregate and cluster to form a barrier for motor neurons but not motor axons. If the BCC clusters fail to form properly, they cannot fulfill this barrier function and motoneurons stream out of the spinal cord along the ventral roots.

## Discussion

Entry and exit sites of the CNS are well controlled transition areas that are permissive for axons but not for cell bodies during development due to the presence of the BCCs. The boundary cap is a transient structure that disappears at postnatal day 6 in the rat [49]. In chicken, BCC clusters labeled by KROX20 or SEMA6A disappear between HH36 and HH40 (Figure 1). They are replaced by a non-permissive barrier at the CNS/PNS interface consisting of astrocytes and Schwann cells [36,49]. BCCs originate from a late-migrating population of neural crest cells [38]. So far, they had been identified only after clustering by their expression of KROX20 and Cadherin-7. A time course of SEMA6A expression analyzed in transverse sections from the lumbosacral region of the embryonic chicken spinal cord suggests that BCCs express SEMA6A while they still migrate toward and cluster at the entry and exit sites of the spinal cord (Figure 1). The confined expression of SEMA6A in boundary cap cells together with the striking observation by Vermeren and colleagues [37] that ablation of BCC clusters resulted in the emigration of motoneurons from the ventral spinal cord into the periphery motivated us to test for a role of Sema6A in BCCs as a gate keeper between the CNS and the PNS. Indeed, we found that knock-down of Sema6A resulted in the same phenotype as ablation of the boundary cap (compare Figure 2 to [37]). In the absence of Sema6A from BCCs, motoneurons left the spinal cord along the ventral roots. This effect was specific for loss of Sema6A function. Downregulation of other class 6 semaphorins did not enhance the number of motoneurons found outside the spinal cord compared to control-treated embryos. The fact that we could detect a phenotype of Sema6D loss of function for the dorsal root entry site but not for the ventral motor exit point further confirms the specificity of our approach. Downregulation of a target gene with long dsRNA was specific and efficient, as shown previously [48,50]. The specificity of downregulation was also corroborated by the use of dsRNA derived from a second non-overlapping fragment of cDNA from the 3' end of SEMA6A (data not shown).

Motoneurons leaving the spinal cord were only found after downregulation of Sema6A, while the effect at the dorsal root entry site was also seen after perturbation of Sema6D function, despite the fact that SEMA6D was expressed in ventral and dorsal BCCs. Similarly, downregulation of PlexinA1 had an effect at both the VMEP and the DREZ; loss of PlexinA4 function had an effect only dorsally. The phenotype observed after perturbation of PlexinA1 and PlexinA4 differed from loss of Sema6A/6D function, consistent with a role of class A plexins as receptors for secreted class 3 semaphorins. In the absence of PlexinA1, DRGs were misplaced along the rostrocaudal axis and they were not clearly segregated from each other (Figure 5). These observations are in agreement with studies reporting a role of plexin/neuropilin complexes in the restriction of neural crest migration to the anterior somite [51,52]. Restricted migration through the anterior somite was shown to be essential for the segmental organization of the PNS [53-55]. Thus, in the absence of PlexinA1, not only did dorsal roots fail to segregate properly, as seen after loss of Sema6A function, but the arrangement of the DRGs was also perturbed.

**Figure 9:**
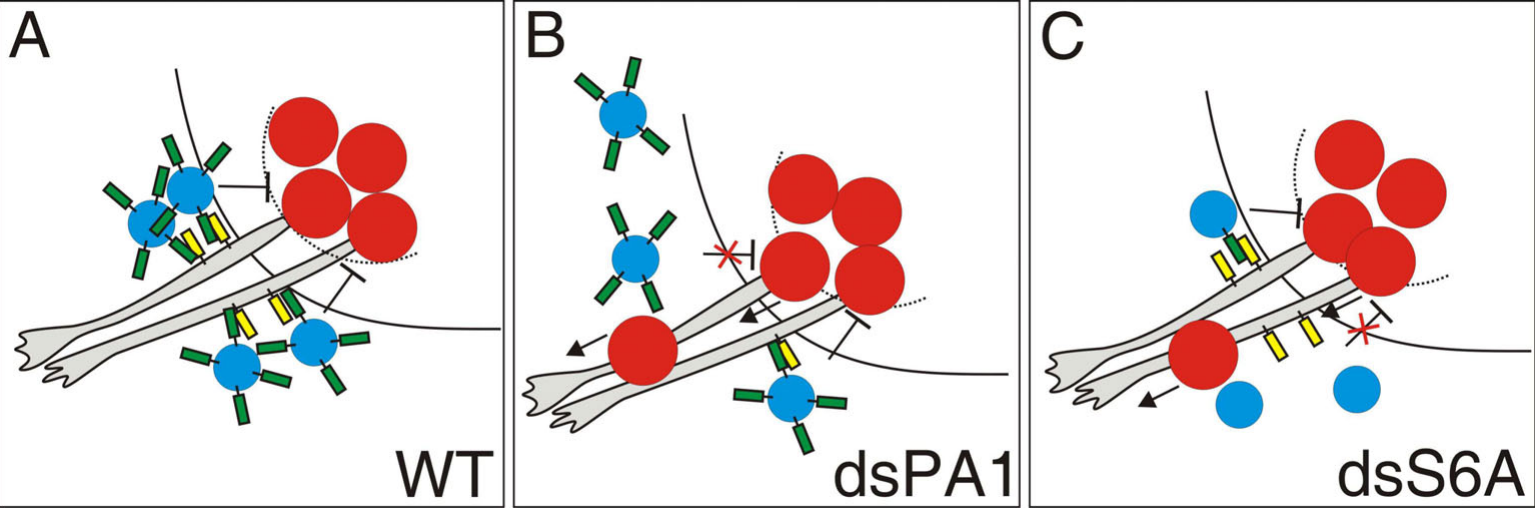
Sema6A acts as a gate keeper at the VMEP by triggering the formation of BCC clusters. Our results support a model that suggests a role for Sema6A in BCC cluster initiation. **(a) **Motor axons leaving the ventral spinal cord express PlexinA1 on their surface (yellow rectangle). Boundary cap cells (blue circles) express Sema6A (green rectangles), which recognizes PlexinA1 on motor axons, resulting in the accumulation of BCCs and, subsequently, in their clustering. By an unknown mechanism the BCC cluster prevents motor neurons (red circles) but not motor axons from translocating into the periphery. **(b, c) **Consistent with this model, the absence of PlexinA1 from motor axons would remove the stop signal (b) and the absence of Sema6A from BCCs would remove the receptor for the stop signal (c). In both cases, BCC clusters would fail to form properly and motoneurons would not be confined to the ventral spinal cord but migrate into the periphery along the ventral roots. The behavior of sensory axons at the dorsal BCC clusters is more complex and cannot be fully explained by this model.

Based on our results, we propose a model where Sema6A in BCCs is required for them to home in on the entry and exit sites of the spinal cord, where they form the boundary cap (Figure 9). PlexinA1 on axons provides the stop signal that is recognized by Sema6A on migrating boundary cap cells. Because we were unable to detect a homophilic Sema6A interaction and none of the PlexinAs is expressed in BCCs, Sema6A is unlikely to be responsible for BCC clustering directly, that is, by mediating cell-cell contact between BCCs. Cadherin-7 is a good candidate for the adhesion molecule that might be responsible for the formation of tight cell-cell contacts between BCCs. Cadherin-7 is expressed strongly when BCCs have reached the aggregation site but not while they are still migrating, and it was shown to bind homophilically [47,56].

According to our model, Sema6A would act as a receptor when expressed in BCCs and recognize PlexinA1 as a ligand. A receptor role for Sema6A has been suggested previously in the brain, where Sema6A was shown to be required for the appropriate targeting of thalamocortical axons [27]. Similarly, a receptor function for Sema6D in neural crest cell migration in heart development was described [23,24]. A receptor function for class 6 semaphorins is also consistent with structural features [26].

Our model is supported by the aberrant clustering of BCCs in the absence of either Sema6A from BCCs or the absence of PlexinA1 from motoneurons (Figure 7). In the absence of Sema6A, BCCs fail to recognize the exit site marked by the first motor axons extending into the periphery, the boundary cap fails to form correctly and, as a consequence, motoneurons are no longer confined to the ventral spinal cord and migrate into the periphery following their axons (Figure 9). The same effect is achieved when PlexinA1 is downregulated in motoneurons. In this case motor axons are unable to provide a stop signal for migrating BCCs. Similarly, the PlexinA1 stop signal can be masked by expression of soluble Sema6A ectodomain or full-length Sema6A in motoneurons. In both cases motor axon-derived Sema6A would compete with Sema6A on the surface of BCCs and result in the aberrant formation of BCC clusters.

In addition to its function as a stop signal for Sema6A-expressing BCCs, PlexinA1 serves as a co-receptor together with neuropilins for class 3 semaphorins. Sema3A was postulated to act as a surround repellent and, thus, to polarize growth of sensory axons during initial stages of development [57]. Later, class 3 semaphorins were shown to interfere with motor and sensory axon pathfinding [2,58-64]. Their effects were mediated by binding to either Neuropilin-1 or Neuropilin-2 associated with one of the class A plexins as the signal transducing part of the receptor.

Chicken embryos express only three PlexinAs, as the gene encoding PlexinA3 is missing from the chicken genome [20]. Similarly, chickens express only three class 6 semaphorins; an ortholog of Sema6C is not found. Therefore, a direct comparison of PlexinA/Sema6 interactions between mouse/human and chicken proteins is not possible. This may explain why, so far, a direct interaction between Sema6A and PlexinA1 has not been demonstrated [25]. The repulsive activity of Sema6A was found to be mediated by PlexinA4 [22,65]. In our *in vivo *assays PlexinA4 had an effect only at the dorsal root entry but not at the ventral motor axon exit site. In our *in vitro *assay, sensory but not motor axons were repelled by Sema6A, despite the fact that all PlexinAs were expressed by sensory and motor neurons [20]. Sema6D was expressed in both dorsal and ventral BCCs but had an effect only at the dorsal root entry zone.

Future experiments will have to elucidate the difference between Sema6A and Sema6D in BCCs and, thus, their different roles in gate keeping between the PNS and the CNS. Obviously, the mechanism differs between the ventral and the dorsal transition zone. Motor axons were not repelled by Sema6A but sensory axons were (Figure 6 and Table 1). The reason for this discrepancy is unknown.

## Conclusion

Sema6A expression by BCCs acts as a gate keeper between the PNS and the CNS by organizing the segregation of dorsal root entry and ventral motor axon exit sites. In both cases Sema6A on BCCs appears to act as a receptor recognizing the stop signal provided by PlexinA1 on axons. As a consequence, BCCs aggregate at the dorsal root entry site and the VMEP. BCCs then form clusters, possibly mediated by Cadherin-7, resulting in a tight barrier that prevents motor neurons from streaming out of the ventral spinal cord along the ventral roots. At the dorsal root entry site the BCCs segregate and organize dorsal roots. Consistent with these observations, Sema6A was found to be a repellent for sensory but not for motor axons.

## Materials and methods

### Cloning of the chicken SEMA6A cDNA

A 728 base-pair fragment of chicken SEMAPHORIN6A obtained in a screen for axon guidance cues [50] was used to screen a λ ZAP library prepared from E14 chicken brains [66]. Two fragments encoding the entire open reading frame (ORF) were ligated and cloned into pBluescript. For the preparation of *in situ *probes and dsRNA we used mainly a fragment spanning the 5' untranslated region and the first 300 base-pairs from the ORF. In addition, we verified the specificity of the phenotype using a fragment from the 3' untranslated region. The alignment of these fragments with SEMA6B and SEMA6D did not result in any significant similarity.

To obtain a soluble AP-tagged ectodomain of Sema6A, the sequence corresponding to the ectodomain of chicken Sema6A (amino acids 1–604) was amplified and inserted into the APtag-2 vector [67]. COS7 cells were transiently transfected with the Sema6A ectodomain-containing plasmid using Lipofectamine 2000 (Invitrogen, Carlsbad, CA). After transfection cells were washed with phosphate-buffered saline (PBS) and grown for 4 days in MEM and 1% fetal calf serum. The supernatant was collected and centrifuged as described in [68]. Binding of AP-tagged Sema6A to cryosections was carried out as described in [69]. Full-length Sema6A with a myc tag was expressed under the control of the β-actin promoter. The plasmid was injected at a concentration of 1 μg/μl into the central canal of the spinal cord of E2.5 embryos followed by electroporation of the ventral spinal cord. The embryos were sacrificed at E5 and analyzed.

### Preparation of *in situ *probes and dsRNA

Probes for *in situ *hybridization and dsRNA were produced from expressed sequence tags (ESTs) obtained from Geneservice Ltd [70]. The ESTs used were: ChEST225N10 (SEMAPHORIN6D), ChEST53D13 and 666O16 (PLEXINA1), ChEST128L21 and 297D11 (PLEXINA2), ChEST1014M19 and 202O14 (PLEXINA4). For SEMAPHORIN6A the cDNA fragment mentioned above was used. For SEMA6B a cDNA fragment was cloned using RT-PCR because no ESTs were available. Total RNA was prepared from HH30 (stage 30 chicken embryos according to Hamburger and Hamilton [42]) spinal cords. Random and oligo dT-primed first-strand cDNAs were generated using Superscript II reverse transcriptase according to the manufacturer's instructions (Invitrogen). A 656 base-pair fragment for SEMA6B was amplified using the antisense primer 5'-CCCATGTCGTTCTTGCAC-3' and the sense primer 5'-ATCCAGCGCATCCTCAAG-3'. The resulting PCR fragments were cloned into the TOPO TA cloning vector (Invitrogen) using *Eco*RI restriction sites (Gemayel *et al*., in preparation). We carefully compared and selected sequences to avoid overlapping stretches that could potentially interfere with RNAi specificity. In fact, off-target effects or unspecific knock-down of related family members were never detected in our approach with long dsRNA, most likely because the concentration of each small interfering RNA produced in a given cell by Dicer is extremely low, with a theoretical maximal concentration of about 1 nM or less [48,50].

*In situ *probes for the detection of KROX20 and SOX10 mRNA were derived from ESTs 738N7 and 477F10, respectively. Plasmid DNA was linearized using restriction enzymes *Not*I, *Eco*RI, *Xba*I, *Hin*dIII, or Asp718 (all from Roche, Basel, Switzerland) to prepare either digoxigenin-labeled *in situ *probes [20] or dsRNA [48] by *in vitro *transcription as described previously.

### *In ovo *RNAi

*In ovo *RNAi was used to knock down genes of interest as described previously [48]. In brief, fertilized eggs were windowed on the second day of incubation to get access to the embryo. Embryos were staged according to Hamburger and Hamilton [42] at the time of injection. A solution containing the dsRNA (200–300 ng/μl) and a plasmid encoding EGFP under the control of the β-actin promoter (50 ng/μl) was injected into the central canal of the spinal cord of HH12-14 embryos to efficiently transfect neural crest cells and motoneurons [71]. The lumbosacral region of the spinal cord was electroporated with 5 pulses of 18 Volts and 50 ms length with a 1 s interpulse interval. Eggs were sealed and put back into the incubator until embryos reached the desired stage. Embryos were sacrificed at HH25 for the analysis of motoneurons and at HH25/26 for the analysis of dorsal roots.

### Tissue preparation

For analysis of phenotypes, embryos were sacrificed, eviscerated and fixed in 4% paraformaldehyde in PBS for 60' to 120' depending on the age. Embryos were rinsed in PBS and subjected to cryoprotection or used directly for whole-mount staining as detailed below. For immunohistochemistry and *in situ *hybridization, the cryoprotected tissue was frozen in isopentane on dry ice and cut into 25 μm thick sections. In situ hybridization was carried out as detailed previously [20]. For immunohistochemistry, the staining protocol described earlier [72] was used. Antibodies were diluted in blocking buffer (10% fetal calf serum in PBS). For permeabilization of the tissue, sections were incubated for 1' in 0.1% Triton-X-100. The antibodies used were: monoclonal antibodies 1E8 recognizing P0, 40.2D6 recognizing Isl-1, and 9E10 recognizing the myc tag (all from the Developmental Studies Hybridoma Bank, University of Iowa, Iowa City, IA) Furthermore, we used rabbit anti-neurofilament (Millipore, Billerica, MA), and a FITC-labeled goat anti-GFP antibody (Rockland, Gilbertsville, PA). Secondary antibodies were: goat anti-mouse IgG Cy3 (Jackson ImmunoResearch Newmarket, Suffolk, UK), goat anti-rabbit Alexa350, and goat anti-rabbit Alexa488 (both Invitrogen/Molecular Probes, Carlsbad, CA).

### Neurofilament staining of whole-mount embryos

For whole-mount staining, embryos were sacrificed at HH25/26, fixed as described above and transferred to 24-well plates. Tissue was permeabilized in 1% Triton/PBS for 1 h at room temperature, rinsed in PBS, and incubated in 20 mM lysine in 0.1 M sodium phosphate (pH 7.3) for another hour. After rinsing thoroughly in PBS, embryos were incubated in blocking buffer (10% fetal calf serum in PBS) for at least two hours before the anti-neurofilament antibody (RMO270 from Zymed/Invitrogen, Carlsbad, CA, diluted 1:1,500) was added for 48 h at 4°C. Incubation with the secondary antibody (goat anti-mouse IgG Cy3, 1:250) was for 12 h. EGFP was visualized with a FITC-labeled goat anti-GFP antibody. Embryos were rinsed thoroughly and dehydrated in a graded series of methanol before transfer to benzyl benzoate/benzyl alcohol (2:1).

### Quantification of the phenotypes

Experimental embryos and control-treated embryos that were injected and electroporated with the plasmid encoding EGFP only were sacrificed at HH25/26. Tissue preparation, cutting and staining was as detailed above. From each embryo lumbosacral sections were analyzed by an observer who was blind to the treatment group. Sections were classified into groups containing either 0–1, or more than one Isl-1-positive cell along the root. All sections that contained EGFP and the ventral roots were analyzed and scored. The percentage of sections per embryo containing more than one motoneuron outside the spinal cord was calculated.

For the analysis of the phenotype at the dorsal root entry site, embryos were sacrificed at HH25/26 and stained with RMO270 and goat anti-mouse IgG Cy3 as whole-mounts as detailed above. For the analysis of the segregation of DRGs and dorsal roots, a dissection microscope equipped with fluorescence optics (Olympus SZX12) was used. Single fibers crossing to the adjacent DRG or irregular spacing was considered a weak phenotype. When roots were formed by sensory axons emanating from two DRGs or when the DRGs were fused, the embryo was scored as having a severe phenotype. For statistical analysis, we used two-way ANOVA with Bonferroni correction. Values represent mean ± standard error of the mean.

### *In vitro *assay

COS7 cells grown on 8-well LabTek slides were transfected with pcDNA3.1 vectors containing myc-tagged SEMA6A, myc-tagged AXONIN-1, or farnesylated EGFP (Invitrogen) as a control using Lipofectamine 2000 (Invitrogen). Sensory and sympathetic ganglia were dissected from HH26 or HH35 embryos. Motoneurons were obtained from the ventral halves of spinal cords dissected from HH26-28 embryos. Single-cell suspensions were obtained by digestion of ganglia and ventral spinal cord junks with trypsin followed by trituration. Per well, 25,000 DRG or sympathetic neurons or twice as many motoneurons were plated. DRG and sympathetic neurons were cultured in serum-free medium containing 20 ng/ml nerve growth factor (NGF) (see [73] for details). Motoneurons were cultured in MEM containing 5% fetal calf serum, N3, and 1 mM sodium pyruvate. Neurons were grown on transfected COS cells for one (DRG, sympathetic neurons) or two days (motoneurons) before fixation in 4% paraformaldehyde for 30 minutes at room temperature and staining with the 9E10 antibody (Developmental Studies Hybridoma Bank) to detect successfully transfected cells and rabbit anti-neurofilament to stain axons.

Cultures were analyzed and the behavior of axons encountering a transfected COS cell was classified as avoidance if an axon stopped or turned away from a transfected cell, or as attraction if an axon failed to leave the surface of a transfected cell. We chose Axonin-1 as a control protein because it was shown to promote axon outgrowth of sensory neurons [73,74]. In addition, Axonin-1 was shown to be required for pathfinding of nociceptive afferents [75] and axons of dorsolateral commissural neurons [48,76] but not for extension of commissural axons [77].

## Competing interests

The author(s) declare that they have no competing interests.

## Authors' contributions

OM, ED, IA, RS, and ES carried out experiments and analyzed the data. OM prepared the figures. ES conceived the study and wrote the manuscript.

## Supplementary Material

Additional file 1 Downregulation of the targeted PlexinA was specific. For each PlexinA we used two different cDNA fragments to produce long dsRNAs for gene silencing (see Materials and methods for details). As shown qualitatively by *in situ *hybridization (a-c, e-g, i-k) and quantitatively by analyzing intensity levels for the three PlexinAs with ImageJ 1.38× (m) downregulation of the targeted PlexinA was specific. Downregulation of PLEXINA1 (PA1; a-d) resulted in a reduction of PA1 expression in motoneurons (arrowhead in (a)). Pattern and expression levels of PA2 (b) and PA4 (c) were not changed. EGFP expression from a co-injected plasmid indicates the electroporated half of the spinal cord (d). Similarly, silencing of PA2 (e-h) resulted in changes of the PA2 expression pattern in motoneurons (arrow in (f)) on the electroporated side (see (h)) but the expression of PA1 (e) and PA4 (g) were not altered. Targeting PA4, which is very weak at HH25, further reduced expression of PA4 in motoneurons (arrowhead in (k)) on the electroporated side (l) but had no effect on the expression of PA1 (i) and PA2 (j). The quantification of the signal intensity is shown in (m). Similar levels were obtained for all PlexinAs. Three or four different embryos were analyzed per condition using at least ten sections. Specific downregulation was 24.9 ± 5.7% for PA1, 26.8 ± 5.5% for PA2, and 25.9 ± 2.1% for PA4. Values are shown ± standard deviation. *P *< 0.0001 indicated by three asterisks. Note that the section shown in (f) was taken from the thoracic level. AS, antisense probe. Click here for file
