# Supplementary figures and images for: Semaphorin6A acts as a gate keeper between the central and the peripheral nervous system
Source: Neural Dev. 2007 Dec 18;2:28. doi: 10.1186/1749-8104-2-28 (PMC2238753; doi:10.1186/1749-8104-2-28)

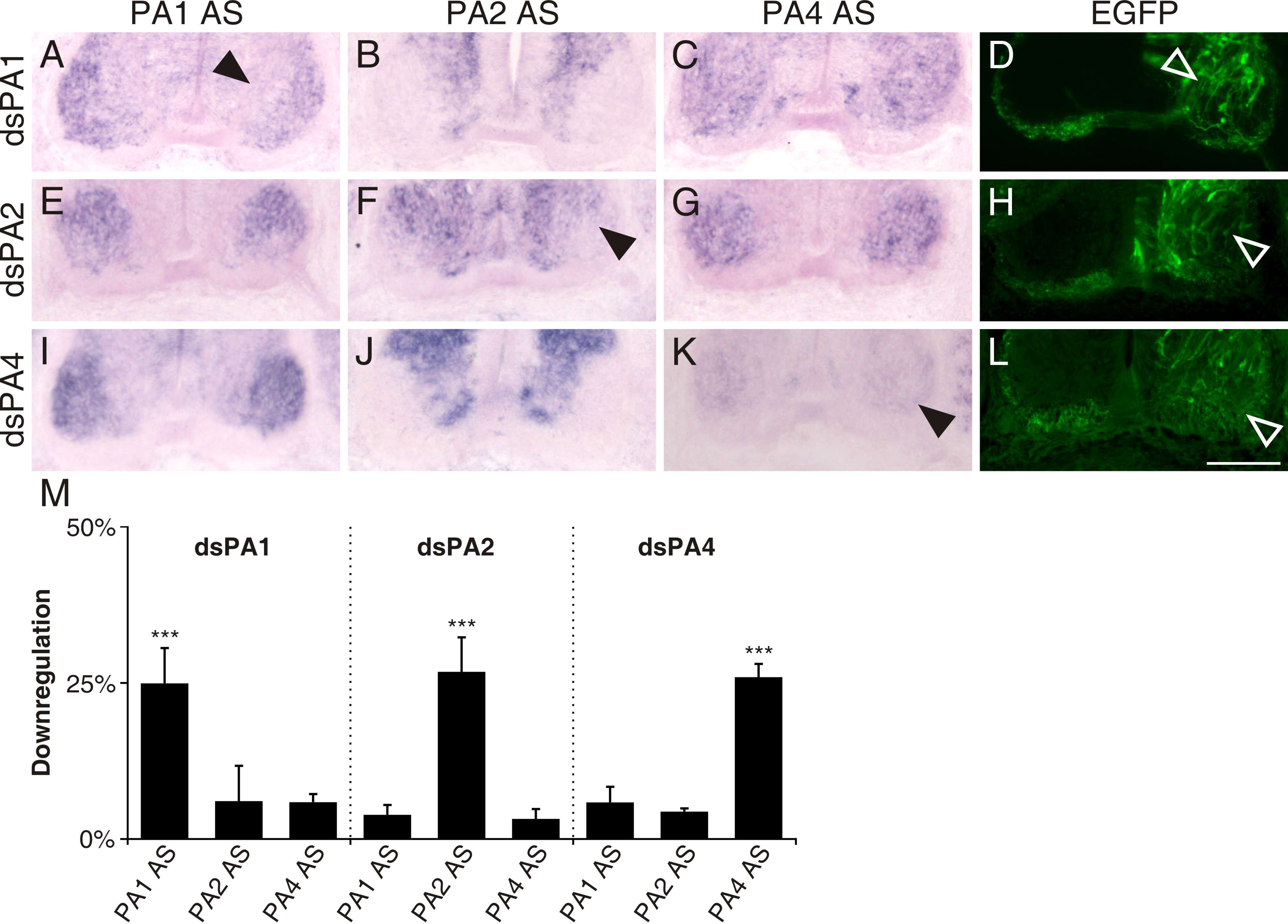

Supplement: Additional file 1 — Downregulation of the targeted PlexinA was specific. For each PlexinA we used two different cDNA fragments to produce long dsRNAs for gene silencing (see Materials and methods for details). As shown qualitatively by in situ hybridization (a-c, e-g, i-k) and quantitatively by analyzing intensity levels for the three PlexinAs with ImageJ 1.38× (m) downregulation of the targeted PlexinA was specific. Downregulation of PLEXINA1 (PA1; a-d) resulted in a reduction of PA1 expression in motoneurons (arrowhead in (a)). Pattern and expression levels of PA2 (b) and PA4 (c) were not changed. EGFP expression from a co-injected plasmid indicates the electroporated half of the spinal cord (d). Similarly, silencing of PA2 (e-h) resulted in changes of the PA2 expression pattern in motoneurons (arrow in (f)) on the electroporated side (see (h)) but the expression of PA1 (e) and PA4 (g) were not altered. Targeting PA4, which is very weak at HH25, further reduced expression of PA4 in motoneurons (arrowhead in (k)) on the electroporated side (l) but had no effect on the expression of PA1 (i) and PA2 (j). The quantification of the signal intensity is shown in (m). Similar levels were obtained for all PlexinAs. Three or four different embryos were analyzed per condition using at least ten sections. Specific downregulation was 24.9 ± 5.7% for PA1, 26.8 ± 5.5% for PA2, and 25.9 ± 2.1% for PA4. Values are shown ± standard deviation. P < 0.0001 indicated by three asterisks. Note that the section shown in (f) was taken from the thoracic level. AS, antisense probe. [file 1749-8104-2-28-S1.jpeg]
